# Editorial: Advances in PET-CT imaging

**DOI:** 10.3389/fmed.2025.1722348

**Published:** 2025-10-29

**Authors:** Francesco Dondi

**Affiliations:** Nuclear Medicine, Università degli Studi di Brescia and ASST Spedali Civili di Brescia, Brescia, Italy

**Keywords:** [^18^F]fluorodesoxyglucose ([^18^F]FDG), non-small cell lung cancer (NSCLC), fibroblast activation protein (FAP) inhibitor (FAPI), prostate specific membrane antigen (PSMA), [^68^Ga]Ga-PSMA-11 PET, multiparametric magnetic resonance imaging (mpMRI), PET/CT

This Research Topic collected 15 articles that underlined the advances of positron emission tomography/computer tomography (PET/CT) imaging both in clinical and preclinical settings. In recent years, the use of PET/CT imaging has grown steadily, demonstrating its added value in the assessment of a wide range of diseases, including for example, neoplasms and inflammatory-infectious conditions. In this setting, the continuous development of different radiopharmaceuticals allows an expansion of the application scope of this imaging modality, providing ongoing new research opportunities and potential future clinical applications.

[^18^F]fluorodesoxyglucose ([^18^F]FDG) is currently the most widely used radiotracer, since its ability to underline the increased glycolytic activity of cells and tissue in different pathological conditions. In this setting, Yu et al. assessed the diagnostic efficacy and necessity of [^18^F]FDG PET/CT in fever of unknown origin, demonstrating sensitivity of 79.5%, specificity of 61.1%, positive predictive value of 75.6% and negative predictive value of 66.3% for the final diagnosis. In addition, in the case of true-positive PET imaging, correlations with localized pain and prolonged activated partial thromboplastin time were found, identifying therefore clinical factors associated with PET positivity.

Blockmans et al. applied [^18^F]FDG PET/CT imaging for the evaluation of giant cell arteritis, revealing that in addition to the extent and the intensity of the initial vascular inflammation, aortic inflammation may contribute to the development of aneurysms of the thoracic aorta.

An interesting case reported by Qi et al. underlined the possible clinical application of [^18^F]FDG PET/CT in the evaluation of Rosai-Dorfman disease, reporting a rare case of unifocal extranodal localization that occurred in the sigmoid. Interestingly, a sort of similar pattern of appearance between the contrast-enhanced CT scan (mild to moderate enhancement) and the PET/CT scan (mild to moderate tracer uptake) was reported. Similarly, Hu, Zhao, Yu, et al. presented an interesting case of a 10-year-old girl diagnosed with inflammatory myofibroblastic tumor of the sigmoid colon, a rare site for its presentation, characterized as a mass with uneven enhancement on contrast-enhanced CT and increased [^18^F]FDG uptake.

Not moving from the oncological setting, a case report by Hu, Li, et al. provided a valuable reference for imaging findings in primary clear cell carcinoma of the liver, a rare subtype of hepatocellular carcinoma, which presented at [^18^F]FDG PET/CT as unevenly low-density with increased tracer uptake, while on contrast-enhanced CT or T1WI it may present significant enhancement. Hu, Zhao, Li, et al. reported an interesting and rare case of a pulmonary mixed squamous cell and glandular papilloma that presented as a lung solid nodule with no lobulation or spiculation but with significant enhancement on contrast-enhanced CT and increased [^18^F]FDG uptake on PET/CT; the subsequent literature review revealed that tracer uptake can range from mild to significant.

Yu and Chen performed a head-to-head comparative meta-analysis to evaluate [^18^F]FDG PET/CT vs. [^18^F]FDG PET/magnetic resonance imaging (MRI) in the staging of non-small cell lung cancer (NSCLC). The sensitivity and specificity for detecting nodal metastases were 0.82 (0.68–0.94) vs. 0.86 (0.70–0.97) and 0.88 (0.76–0.96) vs. 0.90 (0.85–0.94), for the two modalities respectively, while focusing on distant metastases, the values were 0.86 (0.60–1.00) vs. 0.93 (0.63–1.00) and 0.89 (0.65–1.00) vs. 0.90 (0.64–1.00), respectively. No significant differences were reported, showing that the two modalities has similar value for the evaluation of NSCLC localization.

As mentioned, different radiotracers can be used to image different metabolic pathways with PET imaging. Fibroblast activation protein (FAP) inhibitor (FAPI) is used for the assessment of fibroblasts activity. In this setting Dai et al. evaluate the impact of milk consumed prior to PET/CT on [^18^F]AlF-NOTA-FAPI-04 uptake in normal abdominal organs to avoid gallbladder and biliary tract uptake, revealing that there was a reduction in gallbladder uptake in treated group (*p* < 0.001, average SUVmean 2.19 ± 2.01 vs. 10.04 ± 9.66). A subgroup analysis revealed that [^18^F]FAPI uptake of liver and small intestine was significantly lower than [^18^F]FDG uptake in both the treated and control group (*p* < 0.001). The diagnostic yield of FAPI tracers in the evaluation of thyroid cancer was investigated in a systematic review by Rizzo et al.. In a per-patients analysis on the detection of local recurrence and distant metastases of differentiated thyroid cancer, overall detection rate of approximately 85%, overall sensitivity and specificity of 96% and 50% respectively and detection rate for lymph node metastases of 86%, for lung lesions of 81.7% and bone secondaries of 100% were reported. In a per-lesion analysis, sensitivity and specificity for neck lesions were 83% and 42% respectively and detection rate for distant and nodal metastases of 79% and 95.4%, respectively. FAPI imaging was able to reveal a higher number of lesions in lymph nodes, lung, bone and liver compared to [^18^F]FDG, however its superiority was not statistically significant in most of the cases. Additionally, FAPI PET demonstrated high accuracy for detecting local recurrences and distant metastases of medullary thyroid cancer when compared to [^68^Ga]Ga-DOTANOC PET/CT.

The detection of cardiac neuroendocrine tumor metastases with somatostatin receptor PET/CT was evaluate in a meta-analysis by Campanale et al. Compared to other radiological techniques, PET/CT was able to detect earlier these metastases and patients were often asymptomatic and had other disease localization. The pooled prevalence of cardiac metastases of neuroendocrine neoplasm among those performing PET/CT was 1.5% [95% confidence interval (CI) 1.0%−1.9%].

Prostate specific membrane antigen (PSMA) is a radiopharmaceutical typically used for the evaluation of prostate cancer. A meta-analysis comparing [^68^Ga]Ga-PSMA-11 PET and multiparametric magnetic resonance imaging (mpMRI) in the diagnosis of initial lymph node staging was performed by Wang et al. PET imaging demonstrated an overall sensitivity of 73% (95% CI 51%−91%) and an overall specificity of 94% (95% CI 88%−99%), while mpMRI showed a sensitivity of 49% (95% CI 30%−68%) and a specificity of 94% (95% CI 88%−99%). Despite that, the differences in sensitivity and specificity were not statistically significant.

Immuno-PET is an innovative medical imaging technique that combines antibodies or similar immuno-targeting molecules with positron-emitting radionuclides, enabling therefore the detection of specific cells. In this setting, Koenen et al. reported a case series of patients imaged with [^89^Zr]Zr-crefmirlimab berdoxam PET/CT to assess CD8^+^ T-cell localization during active COVID-19 infection. In particular, PET imaging demonstrated the differential distribution of CD8^+^ T-cells in the mucosa and associated lymphoid organs of the upper respiratory tract during early and later stages of the infection, underscoring the concept of spatial and temporal compartmentalization of T-cell responses to respiratory viral infection. Moreover, differences in patterns of CD8^+^ T-cell distribution across mucosa, primary and secondary lymphoid organs, blood pool, and peripheral tissues could be correlated with changes in CD8^+^ T-cell functional phenotypes and had a coincidence with different intensity of tracer uptake. Habte and Natarajan proposed the basis for the initial validation of the potential usage of ultra-low-dose clinical practices using target-specific immuno-PET, by developing a novel immuno-PET with an intact rituximab antibody labeled with [^64^Cu]Cu to image human CD20 in a transgenic mouse model for non-Hodgkin's lymphoma imaging. They underlined that an ultra-minimal dose administered in a mouse model [25 μCi] showed good image quality with high signal-to-noise ratio without compromising quantitative accuracy.

Moving to a more technical field, Metrard et al. proposed an interesting literature review of contrast-enhanced PET/CT, reporting that the use of iodinated contrast enhanced its overall performances by combining sensitivity and specificity of PET with those of diagnostic CT. In addition, improvement in patient dosimetry, facilitating pathology management and decreasing the administered volumes of contrast agent were important factors taken into account. Despite that, side effects related to contrast agents injection may be present and needs therefore to evaluated when assessing their use. Lastly, Thormann et al. compared two different software packages for the evaluation of acute ischemic stroke at perfusion CT (CTP), underscoring significant variability in their ability to reliably rule out small lacunar infarcts. In particular, one of them demonstrated a good specificity, suggesting that dependable CTP-based stroke exclusion is achievable with advanced post-processing.

In conclusion, this Research Topic includes different papers that underline the potential applications of PET/CT in different fields. Different radiotracers will probably constantly gain relevant role for the assessment of several pathological conditions, aiming therefore a more personalized evaluation of the patients.

